# Antiemetic efficacy of single-dose palonosetron and dexamethasone in patients receiving multiple cycles of multiple day-based chemotherapy

**DOI:** 10.1007/s00520-012-1469-9

**Published:** 2012-04-26

**Authors:** Vito Lorusso, Marianna Giampaglia, Luciana Petrucelli, Valeria Saracino, Tania Perrone, Antonio Gnoni

**Affiliations:** 1 Oncology Unit, Vito Fazzi Hospital, Lecce, Italy; 2 Scientific Department, Italfarmaco, Cinisello Balsamo, MI Italy

**Keywords:** Chemotherapy-induced nausea and vomiting (CINV), Food intake, Multiple day chemotherapy, Multiple cycles, Palonosetron

## Abstract

**Introduction:**

The goal of pharmacological prophylaxis of chemotherapy-induced nausea and vomiting (CINV) should be the elimination of both nausea and vomiting symptoms during all planned chemotherapy cycles. The aim of this study was to assess the efficacy of a single dose of palonosetron and dexamethasone to prevent CINV and to guarantee an adequate food intake (FI) in patients receiving several cycles of multiple day-based chemotherapy (MD-CT).

**Methods:**

Patients with advanced cancer but without a compromised nutritional status (bone mass index ≥ 18.5) were treated with 0.25 mg palonosetron plus 20 mg dexamethasone before MD-CT. The MD-CT regimen was either epirubicin plus ifosfamide or paclitaxel plus cisplatin and ifosfamide. Nausea, vomiting, and FI were monitored in a 7-day diary. Complete response (CR: no vomiting and no rescue therapy) was the primary endpoint, while complete control (CC: CR and no more than mild nausea) and the evaluation of FI were secondary endpoints. The endpoints were evaluated during the overall timescale (0–168 h) of the chemotherapy regimen.

**Results:**

Fifty patients were enrolled, 80% of whom achieved CR and 78% achieved CC. During the six chemotherapy cycles, CR and CC ranged from 76% to 88% and from 62% to 88%, respectively. Moreover, patients with CR had a significantly (*p* < 0.0001) higher weekly food intake compared with patients not achieving CR.

**Conclusions:**

This trial was the first to assess the efficacy of palonosetron and dexamethasone for the prevention of both nausea and vomiting in patients receiving multiple cycles of MD-CT. In this trial, the ability of patients to intake an adequate amount of food each week was correlated with nausea, thus providing clinicians with an objective parameter for the measurement of the effects of nausea. A single dose of palonosetron and dexamethasone was able to prevent CINV in most patients receiving 3 days of chemotherapy during all planned chemotherapy cycles.

## Introduction

Prevention, rather than treatment, is the principal aim of antiemetic prophylaxis in patients undergoing chemotherapy [1]. Control of chemotherapy-induced nausea and vomiting (CINV) is necessary to maintain an adequate quality of life and to guarantee the completion of treatment during all planned chemotherapy cycles [1]. Few studies have assessed the efficacy of antiemetic prophylaxis over multiple chemotherapy cycles [2–4]. It is also well-known that patients who are not adequately protected from nausea and vomiting during the first chemotherapy cycle are at a higher risk of developing anticipatory emesis in subsequent cycles [5]. As a consequence, CINV control becomes more difficult.

Risk factors for CINV are both patient- and chemotherapy-related: female gender, young age, motion sickness, and no alcohol consumption are patient risk factors while the emetogenic potential of the chemotherapy drug and multiple day (MD) and/or high dose administration are chemotherapy-related risk factors [6, 7].

Patients treated with MD chemotherapy (MD-CT) are at risk from CINV throughout the entire treatment period because continuous daily emetogenic stimuli make antiemetic prophylaxis quite problematic [7]. For CINV prophylaxis in patients receiving MD-CT, antiemetic guidelines suggest the use of a single dose of palonosetron, a second-generation 5-HT3 receptor antagonist (5-HT3RA), instead of multiple daily doses of first generation 5-HT3RAs (ondansetron, granisetron, tropisetron), with the addition in both cases of dexamethasone [8, 9].

Palonosetron demonstrated superior results in preventing CINV in high (HEC) and moderately (MEC) emetogenic chemotherapy compared with first-generation 5-HT3RAs [10–14]. Indeed, besides controlling vomiting, palonosetron in combination with dexamethasone, as compared with other antiemetic drugs [14–16], is also better at controlling nausea and can guarantee an adequate caloric intake during the 7-day period following the first chemotherapy cycle [17]. In a previous study, we proposed the use of a nutritional diary for the measurement of caloric intake, to more objectively define the impact of nausea on the patient’s quality of life. In our hands, this tool provided an objective measurement of the improvement of patient’s quality of life induced by the pharmacological prophylaxis.

The antiemetic efficacy of palonosetron has routinely been assessed during the first chemotherapy cycle [5, 7]; however, no published clinical trial has evaluated its prospective efficacy over the course of the entire patient treatment plan. However, a recent survey assessed the safety of palonosetron administered over multiple cycles of 1-day MEC and HEC, demonstrating that it was well tolerated, with no unexpected treatment adverse events in later cycles [18].

The aim of this study was to therefore assess the efficacy of a single dose of palonosetron and dexamethasone to prevent CINV, in order to ensure adequate food intake, in patients receiving numerous cycles of MD-CT.

## Patients and methods

The study presented here describes a prospective, uncontrolled trial conducted in the Oncology Department of the Vito Fazzi Hospital in Lecce, Italy. The study was approved by the local ethic committee, and all patients signed the informed consent form.

Eligible cancer patients receiving MD-CT were enrolled in the study. The main inclusion criteria were: age >18 years old; ECOG 0–1; normal renal and liver function; no uncontrolled vomiting; and absence of intestinal obstruction, peritonitis, serious mucositis, and infections.

A nutritional assessment was performed for each patient, and all eligible patients were required to have a moderate or well-nourished nutritional status according to the subjective global assessment (SGA). In addition, a bone mass index (BMI) ≥18.5 was required.

The MD-CT regimen was either EPI-IFO (epirubicin plus ifosfamide) or TIP (paclitaxel plus cisplatin and ifosfamide). Refer to Table 1 for the study treatment flow chart.

**Table 1:** Chemotherapy regimens, antiemetic prophylaxis, and study assessments

Variable	D1	D2	D3	D4	D5	D6	D7
Chemotherapy regimens							
EPI-IFO							
Epirubicin 60 mg/m^2^	X						
Ifosfamide 3 g/m^2^	X	X	X				
TIP							
Paclitaxel 175 mg/m^2^	X						
Cisplatin 75 mg/m^2^		X					
Ifosfamide 1,5 g/m^2^	X	X	X				
Antiemetic prophylaxis							
Palo 0.25 mg	X						
Dex 20 mg	X						
Study evaluations							
CINV	X	X	X	X	X	X	X
FI	X	X	X	X	X	X	X

Abbreviations: *D* day, *Palo* palonosetron, *Dex* dexamethasone, *CINV* chemotherapy-induced nausea and vomiting, *FI* food intake, *EPI-IFO* epirubicine plus ifosfamide, *TIP* paclitaxel, cisplatin, and ifosfamide

Patients received an antiemetic treatment of a single palonosetron bolus, 0.25 mg iv, over 30 s, and a single dose of dexamethasone, 20 mg, both administered 30 min prior to the administration of chemotherapy. The same antiemetic therapy was administered before each planned chemotherapy cycle.

All patients maintained a diary from day 1 until day 7. The study diary was organized with both an emesis and a nutrition-specific section, as reported in a previous publication [17]. In the emesis section, patients recorded each episode of vomiting, nausea experienced, and any use of rescue medication. In the nutritional section, patients had to quote their daily food intake, the time food was consumed, and the amount of each portion eaten. The amount of portions was quantified using pictures of standard portions included in the diary. The diary had to be completed from day 1 (day of first chemotherapy administration) until day 7 (168 h after chemotherapy administration). On day 8, the patient returned the diary to the investigator and discussed with him all entries regarding CINV information. The nutritional expert then reviewed and discussed all nutritional data.

All patients were monitored for a maximum of six chemotherapy cycles.

### Endpoints

Primary and secondary endpoints were evaluated during the 7 days following the first chemotherapy administration: overall phase (0–168 h). The primary endpoint of the study was the percentage of patients with complete response (CR), defined as no vomiting and no use of rescue medication. Secondary endpoints were: complete control (CC), totally control (TC), and impact of nausea severity and CR on weekly food intake. CC was defined as no vomiting, no rescue medication, and no more than mild nausea; TC was defined as no vomiting, no rescue, and no nausea. Severity of nausea was evaluated using a four-point Likert scale ranging from 0 (none) to 3 (severe). Among the CC patients, the impact of residual nausea on weekly food intake was evaluated, comparing the CC patients with mild nausea versus the CC patients with no mild nausea.

All patients who started the first chemotherapy cycle were evaluated throughout all subsequent cycles (maximum six cycles).

Treatment safety was evaluated during all chemotherapy cycles, and all adverse events were recorded and graded according to the common terminology criteria for adverse events (CTCAE) described by the National Cancer Institute, version 4.0. (http://ctep.cancer.gov/forms/CTCAEv4.pdf).

### Statistical analysis

The demographic and clinical–pathological characteristics were summarized by means of descriptive statistics. In general, absolute and relative frequencies were employed to summarize qualitative variables, while arithmetic mean, standard deviation (SD), median, and range were used to summarize quantitative data.

Main and sub-group comparisons were tested using one-way analysis of variance for continuous variables. The relationship between weekly intake (kilocalories) and nausea scores was investigated by means of linear correlation analysis. Due to the supportive and exploratory nature of these comparisons, no adjustments for multiplicity were performed for the multiple comparisons across cycles, endpoints, and sub-groups. Differences were considered to be statistical significant for two-tailed *p* values ≤ 0.05. All statistical computations were carried out using the SAS system version 9.1.3.

## Results

Between July 2008 and January 2010, 50 consecutive patients were enrolled and evaluated. Most participants were female (82%), chemo-naïve (74%), with a diagnosis of soft tissue sarcoma (52%). All patients received at least two consecutive chemotherapy cycles, and half (50%) completed the six planned chemotherapy cycles. None of the patients were severely malnourished; the majority had an adequate appetite (median apVAS 7) and a median BMI of 26 (range, 18.5–30.5). Baseline patient characteristics and nutritional status are reported in Table 2.

**Table 2:** Baseline patient characteristics and nutritional status

Number of patients, 50	
Age, years	
Median (range)	56.8 (33–81)
Gender % (*N*)	
Male	18 (9)
Female	82 (41)
Diagnosis %(*N*)	
Soft tissue sarcoma	52 (26)
Cervix	34 (17)
Bladder	6 (3)
Breast	6 (3)
Lung	2 (1)
Previous Treatment % (*N*)	
Naive	74 (37)
Not naive	26 (13)
Chemotherapy regimen % (*N*)	
Ifosfamide–epirubicin regimens	68 (34)
Cisplatin-based regimen >50 mg/mq	32 (16)
Number of chemotherapy cycles % (*N*)	
1	100 (50)
2	100 (50)
3	94 (47)
4	76 (38)
5	50 (25)
6	50 (25)
Nutritional status at baseline	
BMI value	
Median (range)	26 (18.5–30.5)
apVAS value	
Median (range)	7 (3–10)
SGA, % (*N*)	
A, well nourished	84 (42)
B, moderately nourished	16 (8)
C, severely nourished	0

*BMI* body mass index, *apVAS* visual analogue scale to assess appetite, *SGA* subjective global assessment

**Fig. 1:**
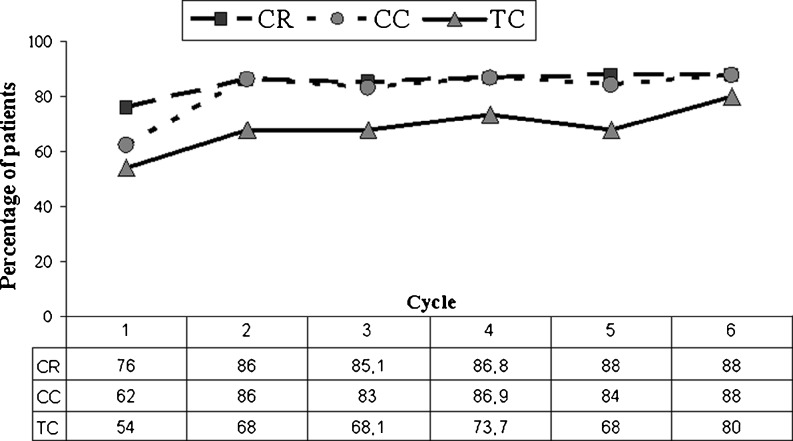
Percentage of patients achieving a complete response (*CR* no vomiting and no rescue medication), a complete control (*CC* no vomiting, no rescue medication and no more than mild nausea), and total control (*TC* no vomiting, no rescue, and no nausea) during the six chemotherapy cycles

**Fig. 2:**
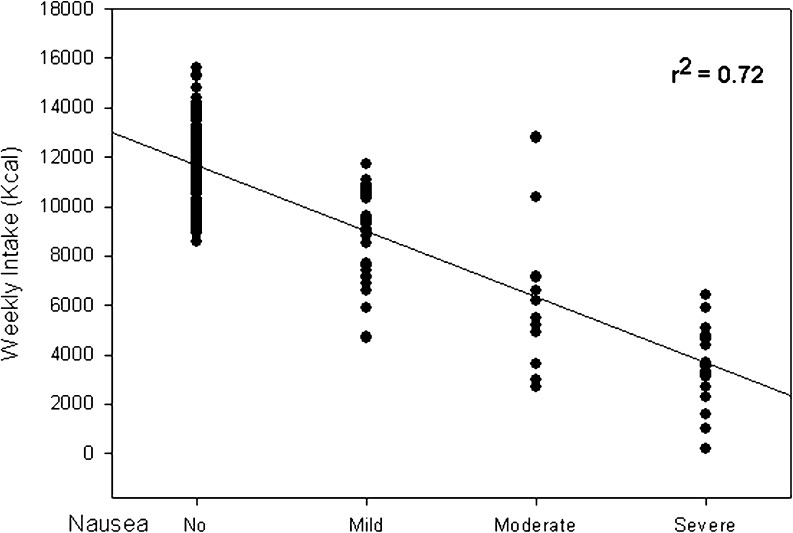
Impact of nausea on food intake during six consecutive chemotherapy cycles

During the six consecutive chemotherapy cycles CR, CC, and TC ranged from 76% to 88%, from 62% to 88% and from 54% to 80%, respectively (Fig. 1). The severity of nausea had a direct impact on food intake (*R* ^2^ = 0.72) (Fig. 2). The percentage of emesis-free patients was 76% (38/50) during the first chemotherapy cycle; 86% (43/50), 89.4% (42/47), 86.8% (33/38) during the second, third, and fourth cycle, respectively; and 88% (22/25) during the final two cycles.

**Fig. 3:**
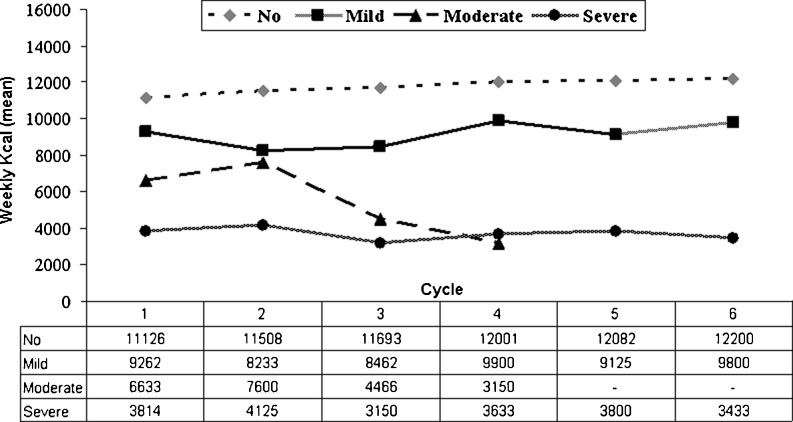
Impact of severity of nausea on weekly food intake during 6 consecutive chemotherapy cycles

The correlation between the severity of nausea and the amount of weekly food intake remained uniform during all subsequent chemotherapy cycles (Fig. 3). During cycles 5 and 6, no patient experienced any nausea of moderate severity.

During all chemotherapy cycles (Table 3), CR patients always had a significantly higher weekly food intake than patients who experienced vomiting and/or used rescue medication (*p* = <0.0001).

**Table 3:** Amount of weekly food intake (expressed in kilocalories) related to complete response during all evaluated chemotherapy cycles

Weekly kilocalories	CR, yes	CR, no	*P* value
Cycle 1, mean ± SD (*N*)	10,725 ± 1,726.49 (38)	5,766.67 ± 3,049.69 (12)	<.0001
Cycle 2, mean ± SD (*N*)	10,927.91 ± 1,962.61 (43)	4,971.43 ± 1,939.69 (7)	<.0001
Cycle 3, mean ± SD (*N*)	11,002.56 ± 1,981.29 (39)	6,314.29 ± 4,486.06 (7)	<.0001
Cycle 4, mean ± SD (*N*)	11,683.33 ± 1,697.55 (33)	3,440 ± 999 (5)	<.0001
Cycle 5, mean ± SD (*N*)	11,577.27 ± 1,770.91 (22)	3,800 ± 556.78 (3)	<.0001
Cycle 6, mean ± SD (*N*)	11,763.64 ± 1,573.07 (11)	3,433.33 ± 1,059.87 (3)	<.0001

*CR* complete response (no vomiting and no use of rescue medication), *SD* standard deviation, *N* number of patients

The impact of mild nausea on food intake was evaluated in the CC population. CC patients who experienced no nausea had a median weekly food intake ranging from 11,102 to 12,200 kcal during multiple chemotherapy cycles, whereas CC patients with symptoms of mild nausea had a median weekly food intake ranging from 8,500 to 9,800 kcal. The difference between the two groups (minimum 2,102 to maximum 2,957 kcal) was statistically significant during all chemotherapy cycles (Table 4). None of the patients experienced mucositis during chemotherapy treatment.

**Table 4:** Impact of mild nausea on weekly food intake among the CC population during the all six chemotherapy cycles

Cycle	Complete control	Weekly kilocalories (mean)	Difference (weekly kilocalories)	*P* value
1	No nausea	11,102	2,602	0.0025
	Mild nausea	8,500		
2	No nausea	11,509	2,776	<0.0001
	Mild nausea	8,733		
3	No nausea	11,632	2,632	0.0001
	Mild nausea	9,000		
4	No nausea	12,002	2,102	0.0085
	Mild nausea	9,900		
5	No nausea	12,082	2,957	0.0010
	Mild nausea	9,125		
6	No nausea	12,200	2,400	0.0431
	Mild nausea	9,800		

## Discussion

The elimination of vomiting and symptoms of nausea should be the goal of antiemetic prophylaxis, not only during the first course of chemotherapy, but also during all planned cycles [1]. Vomiting and nausea still rank among the five most distressing symptoms among cancer patients receiving chemotherapy [19, 20]. In this study, we assessed for the first time the antiemetic efficacy of a single dose of palonosetron and dexamethasone in patients receiving multiple cycles of MD-CT.

For patients receiving MD-CT, the control of CINV is more difficult to achieve due to continuous daily emetic stimuli, especially since acute and delayed emesis may overlap. Antiemetic guidelines for the treatment of MD-CT suggest the administration of a 5-HT3RA once daily at the time of chemotherapy administration and for 2–3 days following the end of treatment. A single intravenous palonosetron dose could be used instead of multiple daily doses of old generation 5-HT3RAs [7, 8]. This was suggested following recent clinical trials, which assessed the efficacy of single and multiple doses of palonosetron both in oncological [17, 21, 22] and hematological [23] MD-CT settings. It has previously been demonstrated that the use of palonosetron can also reduce the use of dexamethasone in antiemetic prophylaxis in patients receiving MEC and AC-based (doxorubicin and cyclophosphamide) chemotherapy [24, 25]. In our trial, the administration of dexamethasone was limited to day 1 only. The reduction of dexamethasone dosing for the prevention of CINV is of particular clinical interest since it has been demonstrated that side effects related to steroid use are likely to accumulate over multiple cycles of chemotherapy [26].

The results of our study suggest that a single dose of palonosetron plus dexamethasone can achieve a high control of CINV during all planned MD-CT cycles, which also confirms the efficacy of palonosetron in maintaining an adequate food intake during all courses. Recently, a survey identified fatigue, appetite loss, and nausea as the most distressing symptoms experienced by cancer patients [20]. These symptoms persist throughout the treatment period and are reported earlier and more frequently by patients than clinicians. In this survey, the cumulative incidence of moderate-to-severe nausea is similar to that of moderate-to-severe appetite loss, suggesting a strict correlation between these two adverse events [20]. In our trial, the correlation between severity of nausea and weekly caloric intake is maintained during all chemotherapy cycles. The patients’ ability to enjoy a meal, even during the week of chemotherapy administration, is necessary to relieve symptoms and to guarantee the completion of the planned cancer treatment. The administration of first-generation 5HT3RAs together with dexamethasone has improved the control of vomiting, and their introduction into routine oncology practices was a key advance, along with other supportive care, towards a major shift in oncology care in the ambulatory setting [27]. However, the control of nausea still remains suboptimal and the impact of residual nausea on patients’ health and quality of life should not be underestimated. Our study (Table 4) highlighted that residual episodes of nausea—no matter how mild—significantly affected the patients’ nutritional status as their weekly food intake was reduced throughout the entire chemotherapy treatment. Indeed, the control of nausea still remains an unmet need in cancer patients receiving chemotherapy.

The role of palonosetron in antiemetic MD-CT prophylaxis, where acute and delayed CINV overlap, is probably explained by its unique pharmacological properties. Palonosetron has a longer half-life (40 h) [28] and a greater receptor binding affinity (>30-fold) than other 5-HT3RAs [29]. Moreover, palonosetron exhibits allosteric interactions [30], triggers receptor internalization, and exhibits prolonged inhibition of receptor function [31]. Palonosetron is the only antiemetic drug that has been demonstrated to ensure an adequate amount of weekly food intake during all chemotherapy cycles due to its ability to control nausea besides vomiting.

Larger and randomized studies are granted to define the role of nausea and vomiting on ability of patients to intake an adequate amount of food during chemotherapy administration, both in MD- and single day-CT.

In conclusion, this study demonstrates that palonosetron and dexamethasone are effective in the prevention of both vomiting and nausea in patients receiving multiple day-based chemotherapy and that this effect is sustained throughout the entire chemotherapy treatment program.
